# *Toxoplasma gondii* chitinase-like protein TgCLP1 regulates the parasite cyst burden

**DOI:** 10.3389/fcimb.2024.1359888

**Published:** 2024-05-17

**Authors:** Hironori Bando, Yuho Murata, Yongmei Han, Tatsuki Sugi, Yasuhiro Fukuda, David J. Bzik, Barbara A. Fox, Kentaro Kato

**Affiliations:** ^1^ Laboratory of Sustainable Animal Environment, Graduate School of Agricultural Science, Tohoku University, Osaki, Miyagi, Japan; ^2^ Department of Parasitology, Asahikawa Medical University, Asahikawa, Hokkaido, Japan; ^3^ National Research Center for Protozoan Diseases, Obihiro University of Agriculture and Veterinary Medicine, Obihiro, Hokkaido, Japan; ^4^ Division of Collaboration and Education, International Institute for Zoonosis Control, Hokkaido University, Sapporo, Japan; ^5^ Department of Microbiology and Immunology, The Geisel School of Medicine at Dartmouth, Lebanon, NH, United States

**Keywords:** toxoplasma, bradyzoite, cyst burden, chitinase, secretory proteins

## Abstract

*Toxoplasma*, an important intracellular parasite of humans and animals, causes life-threatening toxoplasmosis in immunocompromised individuals. Although *Toxoplasma* secretory proteins during acute infection (tachyzoite, which divides rapidly and causes inflammation) have been extensively characterized, those involved in chronic infection (bradyzoite, which divides slowly and is surrounded by a cyst wall) remain uncertain. Regulation of the cyst wall is essential to the parasite life cycle, and polysaccharides, such as chitin, in the cyst wall are necessary to sustain latent infection. *Toxoplasma* secretory proteins during the bradyzoite stage may have important roles in regulating the cyst wall via polysaccharides. Here, we focused on characterizing the hypothetical *T. gondii* chitinase, chitinase-like protein 1 (TgCLP1). We found that the chitinase-like domain containing TgCLP1 is partially present in the bradyzoite microneme and confirmed, albeit partially, its previous identification in the tachyzoite microneme. Furthermore, although parasites lacking TgCLP1 could convert from tachyzoites to bradyzoites and make an intact cyst wall, they failed to convert from bradyzoites to tachyzoites, indicating that TgCLP1 is necessary for bradyzoite reactivation. Taken together, our findings deepen our understanding of the molecular basis of recrudescence and could contribute to the development of novel strategies for the control of toxoplasmosis.

## Introduction

Toxoplasmosis is a zoonotic disease caused by *Toxoplasma gondii*, an obligate intracellular protozoan parasite (Boothroyd, 2009; Dubey, 2010). Although the percentage of seropositive individuals varies by country and dietary habits, it is estimated that more than 30% of the world’s population is infected with *T. gondii* (El-On and Peiser, 2003; Pappas et al., 2009). In most cases, *T. gondii* infection is asymptomatic; however, in the immunocompromised individuals, it leads to severe symptoms, such as hepatitis, encephalitis, and myocarditis (Schlüter and Barragan, 2019; Loeches Yagüe et al., 2023). In addition, parasite infection can cause severe congenital disease in newborn babies born from individuals who contracted the infection for the first time during pregnancy (Weitberg et al., 1979; Frenkel and Remington, 1980; Montoya and Remington, 2008). Furthermore, *T. gondii* is one of the top five human pathogens that causes economic losses and quality-of-life impairment via foodborne illness in the United States (Batz et al., 2012). Therefore, *T. gondii* is an important global zoonotic pathogen.

Within intermediate hosts, which include all warm-blooded animals apart from members of Felidae, *T. gondii* tachyzoites spread through the whole body and infect various organs during acute infection (Dubey, 2009). Although the parasite is eliminated from most organs by host innate immunity (Macmicking, 2012; Gazzinelli et al., 2014), *T. gondii* persists in specific organs, such as muscles and brain, and differentiates from tachyzoites into bradyzoites to cause chronic infection and form cysts (Robert-Gangneux and Darde, 2012; Watts et al., 2015). Bradyzoite cysts remain in the body throughout the host’s life because there are no therapeutic options against the cysts, and they can be reactivated upon host immune depression. In addition, treatment with existing anti-toxoplasmosis drugs, such as pyrimethamine, sulfadiazine, and atovaquone, leads to parasite conversion from tachyzoites to bradyzoites, even though the drugs effectively inhibit tachyzoite growth (Ferguson et al., 1994; Gormley et al., 1998; Alday and Doggett, 2017). Furthermore, it has recently been revealed that chronic infection with *T. gondii* in otherwise healthy individuals may cause other diseases, including depression (Sutterland et al., 2015; Cheng et al., 2020). Given the health and economic burden posed by *T. gondii*, next-generation drugs that can regulate chronic infection are needed.

*T. gondii* secretes various proteins from secretory organelles—namely, micronemes, dense granules, and rhoptries—into host cells to promote efficient intracellular parasite growth and spread infection in the host (Hunter and Sibley, 2012; Hakimi et al., 2017; Bando et al., 2018a). The functions and virulence mechanisms of these proteins during the tachyzoite stage have been extensively analyzed. For example, the microneme proteins (MICs) MIC1, MIC4, and MIC6 form a complex and play an important role in parasite attachment to host cells (Costa Mendonça-Natividade et al., 2020a, b; Zhu et al., 2021). Apical membrane antigen-1 (AMA1), another microneme protein, is involved in intracellular invasion (Santos et al., 2011). Dense granule proteins (GRAs), such as GRA6, GRA7, GRA14, and GRA15, activate host immune responses and the inflammasome (Gov et al., 2013; Gorfu et al., 2014). *T. gondii* inhibitor of STAT1-dependent transcription (TgIST), another dense granule protein, suppresses the activation of host innate immune responses by blocking STAT1 signaling (Olias et al., 2016). Rhoptry bulb proteins (ROPs) ROP5, ROP17, and ROP18 form a complex and protect parasitophorous vacuoles (PVs) from the recruitment of interferon-dependent anti-parasitic molecules like IRG and GBPs (Fentress et al., 2010; Steinfeldt et al., 2010; Behnke et al., 2011; Reese et al., 2011; Rosowski and Saeij, 2012; Jensen et al., 2013; Alaganan et al., 2014; Etheridge et al., 2014; Rosowski et al., 2014; Gay et al., 2016; Olias et al., 2016). ROP2 forms the AMA1–ROP2 complex, which has an important role in intracellular invasion (Roozbehani et al., 2018).

In contrast to the proteins used in the tachyzoite stage, the secretory proteins produced in the bradyzoite stage have not been extensively analyzed. For example, the functions of bradyzoite-secreted proteins such as MIC3 and bradyzoite rhoptry protein 1 (BRP1) are unclear because parasites deficient for these proteins did not show a phenotype (Cérède et al., 2002; Schwarz et al., 2005). The products of a family of *T. gondii* genes related to GRA12 are secreted in both the tachyzoite and bradyzoite stage, and these proteins have been linked to cyst burden and cyst reactivation; however, their mechanisms of action remain unknown (Guevara et al., 2021). In addition, although more than 100 putative bradyzoite-secreted effectors were recently identified by transcriptome analysis, only the cyst wall components BPK1 and MCP4 were characterized and most of the putative bradyzoite-secreted effectors remain uncharacterized (Buchholz et al., 2011). Analyses of the proteins secreted during the bradyzoite stage are important to facilitate the development of novel anti-toxoplasma curative drugs.

Here, we focused on chitinase-like protein 1 (CLP1; TGME49_293770). Although TgCLP1 is secreted in both the tachyzoite and bradyzoite stages, we could not find any notable role for TgCLP1 in the tachyzoite stage. In addition, TgCLP1 did not affect cyst formation. In contrast, we found that TgCLP1 has an important role in bradyzoite reactivation. Our study thus identified a novel protein involved in the cyst burden.

## Materials and methods

### Mice, cells and parasites

BALB/c mice were obtained from Japan SLC. Vero cells (RIKEN BioResource Research Center: RCB0001) were maintained in Dulbecco’s modified Eagle’s medium (DMEM; Nacalai Tesque) containing 10% heat-inactivated fetal bovine serum (FBS; JRH Bioscience), 100 U/mL penicillin, and 0.1 mg/mL streptomycin. HFFs (ATCC; SCRC-1041) were maintained in RPMI (Nacalai Tesque) supplemented with 2% heat-inactivated FBS, 100 U/mL penicillin, and 0.1 mg/mL streptomycin (Nacalai Tesque).

*T. gondii* strain PruΔ*ku80*Δ*hxgprt* or PruΔ*ku80*Δ*hxgprt* LDH2-GFP, which expresses GFP under the control of a bradyzoite-specific gene LDH2 promoter (Fox et al., 2011) was maintained in Vero cells in DMEM containing 10% heat-inactivated FBS (JRH Bioscience), 100 U/mL penicillin, and 0.1 mg/mL streptomycin, as previously described (Bando et al., 2019) and was used as a parental strain for protein tagging and gene disruption.

### Reagents

An antibody against rabbit anti-MIC2-associated protein (M2AP) was kindly provided by Dr. Vern B. Carruthers (John Hopkins University, Baltimore, MD, USA). Mouse anti-CST1 (SalmonE) was kindly provided by Dr. Louis M. Weiss (Albert Einstein College of Medicine, Bronx, NY, USA). DBA-FITC was obtained from EY Laboratories. Succinylated wheat germ agglutinin (sWGA)-FITC was obtained from Vector Laboratories. Anti-SAG1 monoclonal antibody (mAb; TP3) was obtained from HyTest. Anti-GAP45 polyclonal antibody (pAb) was kindly provided by Dr. Dominique Soldati-Favre (University of Geneva, Geneva, Switzerland). Anti-GFP pAb was obtained from MBL.

### *In vitro* bradyzoite differentiation assay

*In vitro* bradyzoite differentiation was performed as previously described (Bando et al., 2021). Briefly, confluent HFFs were infected with tachyzoites and incubated in DMEM (pH 7.2) supplemented with 5% FBS, L-glutamine, penicillin, and streptomycin (normal media) with 5% CO_2_. Three hours post-infection, wells were washed with PBS and cultured in DMEM adjusted to pH 8.3 with 25 mM HEPES, 1% FBS, and penicillin–streptomycin (induction media) for 3–7 days without CO_2_ supplementation.

### Generation of recombinant TgCLP1 protein

RNA was extracted using Trizol (Life Technologies) and isolated with the SV Total RNA Isolation System (Promega) according to the manufacturer’s protocol. Then, the cDNA library was created by using SuperScript III Reverse Transcriptase (Invitrogen). The C-terminal 900 bp of the TgCLP1 coding sequence were amplified by polymerase chain reaction (PCR) from cDNA from the Pru*Δku80Δhxgprt* strain and inserted into the pGEX-6P-2 vector. BL21 competent *Escherichia coli* were transformed with the TgCLP1-fused vector and grown in LB medium supplemented with 50 μg/mL ampicillin for 3 h at 37°C, then IPTG (final concentration: 1 mM) was added and incubated for 16 h at 28°C. The cells were centrifuged at 1,100 ×*g* for 5 minutes, resuspended in sonication buffer (Triton X-100 diluted 1:100 in PBS), and sonicated on ice (30 s of sonication followed by 30 s on ice, repeated 8 times). GST tag-fused recombinant CLP1 was expressed in *E. coli* and purified by GST pull-down using Glutathione Sepharose 4B (GE Healthcare) according to the manufacturer’s protocol.

### Production of polyclonal anti-TgCLP1 antibody

The recombinant protein (50 μg) was emulsified in an equal volume of TiterMax Gold (TiterMax) and subcutaneously injected into 6-week-old female BALB/c mice three times every week. Antisera were collected 4 weeks after the first immunization and used for western blotting.

### Generation of TgCLP1-HA or Myc-TgCLP1-HA parasites

Genomic DNA was isolated from the Pru*Δku80Δhxgprt* or Pru*Δku80Δhxgprt* LDH2-GFP strain with a QIAamp DNA Mini Kit (QIAGEN). To generate TgCLP1-HA or Myc-TgCLP1-HA parasites, 2 kb of the TgCLP1 sequence from the 3’ end were amplified by PCR and cloned into the pMini.ht. C-HA vector between the *Eco*RI and *Eco*RV sites (pTgCLP1-HA). Then, a Myc tag was added to the pMini.ht. C-HA vector (pMyc-TgCLP1-HA). pTgCLP1-HA or pMyc-TgCLP1-HA was linearized, and then transfected as previously described (Bando et al., 2018b). *T. gondii* tachyzoites were transfected with the linearized vector by electroporation. TgCLP1-HA or Myc-TgCLP1-HA parasites were selected based on the presence of a selectable marker, the hypoxanthine-xanthine-guanine phosphoribosyltransferase (HXGPRT) cassette, in DMEM containing 20 µg/mL mycophenolic acid and 50 µg/mL xanthine. Selected parasites were cloned by limiting dilution, and integration of the vector at the TgCLP1 locus was verified by PCR.

### Western blot analysis

Tachyzoites were obtained from infected cells by syringe lysis and passage through a filter (5 µM). Bradyzoites were obtained by syringe lysis of parasite-infected cells that had been prepared as described above. The parasites were centrifuged at 2,000 ×*g* for 5 minutes, then the pellets were resuspended in sodium dodecyl sulfate (SDS) sample buffer after washing with PBS, and boiled for 5 minutes. The samples were separated by SDS–polyacrylamide gel electrophoresis and transferred to a polyvinylidene difluoride membrane for protein blotting (Bio-RAD). The membranes were blocked with Blocking One reagent (Nacalai Tesque), following the manufacturer’s instructions, and incubated with the primary antibodies anti-HA (3F10; Roche), anti-BAG1, anti-ALD1, or anti-TgCLP1 in 5% Blocking One reagent in PBS (BO-PBS) overnight at 4°C. After they were washed three times with 0.1% Tween in PBS, the membranes were incubated with horseradish peroxidase-conjugated goat anti-rat, donkey anti-rabbit, or sheep anti-mouse antibodies (GE Healthcare) for 1 h at room temperature.

### Immunofluorescence assay

Infected cells were fixed on coverslips with 4% paraformaldehyde in phosphate buffer (PFA) for 30 minutes at 4°C and permeabilized with 0.2% Triton X-100, 0.1% glycine, and 0.2% bovine serum albumin in PBS for 5 minutes at room temperature and then blocked with 8% FBS in PBS for 1 h at room temperature. Next, the cells were incubated with the indicated primary antibodies for 1 h at 37°C, followed by incubation with secondary antibodies (Alexa 488- or Alexa 546-conjugated goat anti-rat, anti-mouse, or anti-rabbit IgG; Invitrogen) and DAPI. Coverslips were mounted with Fluorescence Mounting Medium (Dako). Photomicrographs were taken with a TCS SP5 confocal microscope (Leica) or BZ-X800 All-in-One Fluorescence Microscope (KEYENCE).

### Generation of TgCLP1 knock-out parasites by use of CRISPR/Cas9 genome editing

The plasmid pSAG1::Cas9-U6::sgUPRT, which encodes the Cas9 nuclease (GFP fusion) under the control of the *T. gondii* SAG1 promoter, was obtained from Addgene (plasmid 54467). The TgCLP1-targeting CRISPR/Cas9 plasmid (pSAG1::Cas9-U6::sgTgCLP-1) was constructed as previously described (Bando et al., 2018b). To obtain the targeting vector, we amplified 2 kb of the upstream and downstream sequences of the TgCLP1 gene from the genomic DNA of Pru*Δku80Δhxgprt* LDH2-GFP strain and the drug-resistant marker HXGPRT cassette was amplified from the pMini.ht.C-HA vector. These fragments were fused into the pBluescript KS (-) vector with the In-Fusion HD Cloning Kit (Clontech). Before transfection, the vector was linearized with *Spe*I and *Kpn*I. *T. gondii* tachyzoites were transfected with the linearized vector using Basic Parasite Nucleofector Kit 1 and protocol U-030 (Lonza). TgCLP1 knock-out (TgCLP1-KO) parasites were selected based on the presence of the HXGPRT cassette, as described above. Selected parasites were cloned by limiting dilution, and integration of the vector at the TgCLP1 locus was verified by PCR. Deletion of the TgCLP1 protein was confirmed by western blotting with anti-TgCLP1 mouse antisera.

### Complementation of the TgCLP1-KO parasite

Two plasmids were used to generate the TgCLP1 complement plasmid. Plasmid I was pSAG1::cas9-U6::UPRT. Plasmid II was the pBluescript-UPRT vector containing the whole TgCLP1 coding sequence amplified by PCR from the cDNA of the Pru*Δku80Δhxgprt* LDH2-GFP strain. After PCR and sequence checking, these plasmids were transfected into the TgCLP1-KO parasite. To select TgCLP1 complemented parasites, the transfected parasites were cultured with medium containing 5-fluorouracil (final concentration: 5 *µ*M). After three passages, parasites were cloned by limiting dilution. Integration of the vector at theTgCLP1 locus was verified by PCR. Expression of the TgCLP1 protein was confirmed by western blotting with anti-TgCLP1 mouse antisera.

### *T. gondii* invasion assay

The invasion assay was performed as previously described (Bando et al., 2018a). Briefly, tachyzoites purified from host cells were inoculated into HFFs in 12-well plates containing coverslips. The plates were incubated at 4°C for 15 minutes and then 37°C for 3 h to allow parasite invasion. After incubation, the cells were fixed with 4% PFA. A standard non-permeabilized immunofluorescence assay (IFA) was performed by incubating the extracellular parasites with an anti-SAG1 mAb, and then the cells were permeabilized as described above and incubated with the anti-GAP45 pAb. After being washed with 0.1% Tween in PBS, the cells were incubated with secondary antibodies: Alexa 546-conjugated goat anti-mouse IgG for extracellular parasites and Alexa 488-conjugated goat anti-mouse IgG for intracellular parasites. For each experiment, at least 100 parasites were counted and the numbers of extracellular parasites (red) and intracellular parasites (green) were calculated.

### *T. gondii* intracellular replication assay

The intracellular replication assay was performed as described previously (Bando et al., 2019). Briefly, freshly egressed parasites were allowed to invade HFFs grown in 12-well plates containing coverslips for 1 h. The wells were then washed with normal medium to remove extracellular parasites. The parasites were allowed to grow for 24 h at 37°C and then the cells were fixed with 4% PFA. An IFA using anti-GAP45 pAb was performed to stain the intracellular parasites, and the number of parasites per vacuole was scored. For each condition, 100 vacuoles were counted in three independent replicates.

### Plaque assay and measurement of parasitophorous vacuole size

The plaque assay was performed as described previously (Bando et al., 2021). Briefly, confluent monolayers of HFFs grown in 6-well plates were infected with 100 parasites/well and incubated for 8 days at 37°C in 5% CO_2_. Then, the cells were washed twice with PBS, fixed with 2.5% glutaraldehyde–2% paraformaldehyde in phosphate buffer, fixed with 8% PFA, and stained as described above. After they were washed three times, the cells were stained with 0.1% crystal violet at room temperature for 10 minutes. Plaque size was analyzed using Image J. PV size was measured using a BZ-X800 All-in-One Fluorescence Microscope (KEYENCE).

### Bradyzoite reactivation assay

To induce *in vitro* bradyzoite differentiation, parasites were grown in induction medium as described above. After 4 days, the incubation medium was changed and the *T. gondii* was incubated for 24 h in normal medium to induce bradyzoite reactivation. Then, the cells were fixed with 4% PFA and permeabilized as described above for IFA. Tachyzoites were stained with anti-SAG1 mAb (red). Bradyzoites were stained with α-GFP mAb (green). RFP-positive parasites are defined as reactivated parasites. GFP-positive parasites are defined as non-reactivated parasites. The cyst wall was stained with α-CST mAb. To measure the rate of non-reactivated parasites, at least 100 vacuoles were counted, and the numbers of GFP-positive parasites per CST1-positive vacuoles were calculated.

### Assessment of *in vivo* virulence in mice

Mice were intraperitoneally infected with 2.0 × 10^3^ *T. gondii* tachyzoites in 200 μL of PBS per mouse. Survival rates (25 days) and weight (10 days) were measured daily.

### Statistical analysis

All statistical analyses were performed using Prism 7 (GraphPad) or Excel (Microsoft). All experimental points and n values represent the average of three biological replicates (three independent experiments). The statistical significance of differences in mean values was analyzed by using an unpaired two-tailed Student’s t-test. *P* values less than 0.05 were considered statistically significant. The statistical significance of differences in survival times of mice between two groups was analyzed by using the Kaplan-Meier survival analysis log-rank test.

## Results

### The expression patterns of four previously uncharacterized bradyzoite-secreted proteins

**Figure 1:**
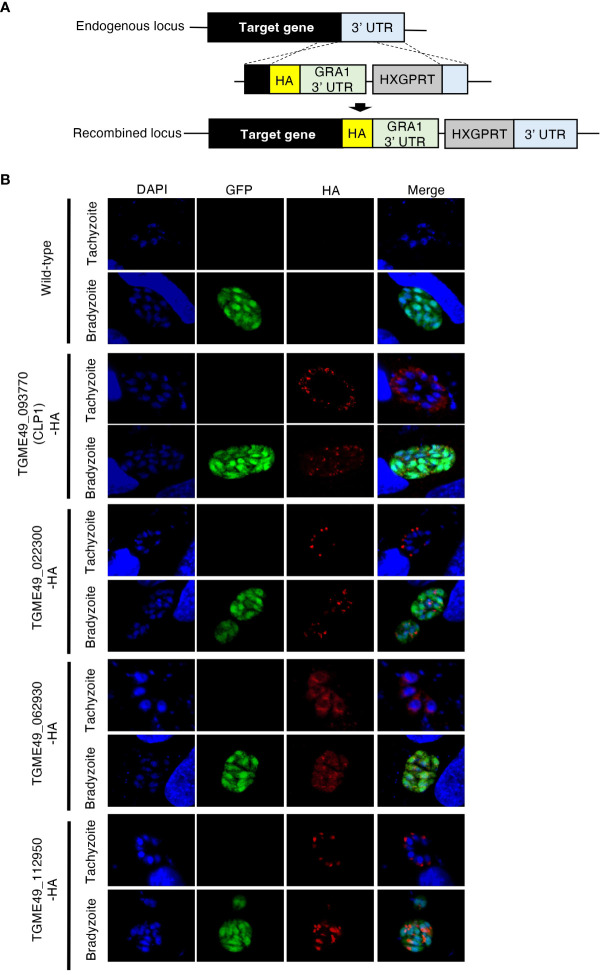
Expression patterns of four uncharacterized proteins. **(A)** Scheme for generating *T. gondii* with a C-terminal HA-tagged target protein using PruΔ*ku80*Δ*hxgprt* LDH2-GFP strain. The construct for inserting the HA-tag is integrated into the genomic sequence of each target gene through single-homologous recombination. **(B)** *T. gondii* with a C-terminal HA-tagged target protein was incubated in normal medium (for tachyzoites) or induction medium (for bradyzoites). The expression of HA-tagged target proteins was detected by IFA. DAPI, nucleus (blue); GFP, bradyzoite marker (green); HA, target protein (red).

A previous study predicted the existence of hundreds of bradyzoite-secreted proteins based on a transcriptomic analysis of *in vivo* and *in vitro T. gondii* bradyzoites (Buchholz et al., 2011). However, analyses of the protein expression patterns, localization, and functions of these proteins have not been conducted. Then, we performed localization analyses by selecting 14 genes with high expression levels in bradyzoite. As a result, we successfully generated four kinds of genetically modified parasites. Thus, we focused on four uncharacterized bradyzoite-secreted proteins: gene accession numbers TGME49_093770, TGME49_022330, TGME49_062930, and TGME49_112950. To determine the expression patterns of these proteins, we inserted an HA-epitope tag endogenously into the C-terminus of each target gene using *T. gondii* type II strain PruΔ*ku80*Δ*hxgprt* LDH2-GFP (**Figure 1A**). Then, the expression of these C-terminal HA-tagged proteins in tachyzoites and bradyzoites was analyzed (**Figure 1B**). We first confirmed that the proteins encoded by TGME49_022330, TGME49_062930, and TGME49_112950 were expressed in both the tachyzoite and bradyzoite stages (**Figure 1B**). TGME49_093770, also called *T. gondii* chitinase-like protein 1 (TgCLP1), has previously been detected in only tachyzoites (Huynh et al., 2009); however, we confirmed that it is also expressed in bradyzoites (**Figure 1B**). The properties of TGME49_022330, TGME49_062930, and TGME49_112950 are largely unknown, whereas TgCLP1 has a chitinase-like domain. Previous studies suggest that an interaction between the *T. gondii* cyst wall, which contains chitin, and host chitinase is important to control chronic infection (Nance et al., 2012). Therefore, we hypothesized that TgCLP1 has an important role in the parasite life cycle, especially in chronic infection and focused it in this study.

### C-terminal HA-tagged TgCLP1 localizes to the microneme in both tachyzoites and bradyzoites

**Figure 2:**
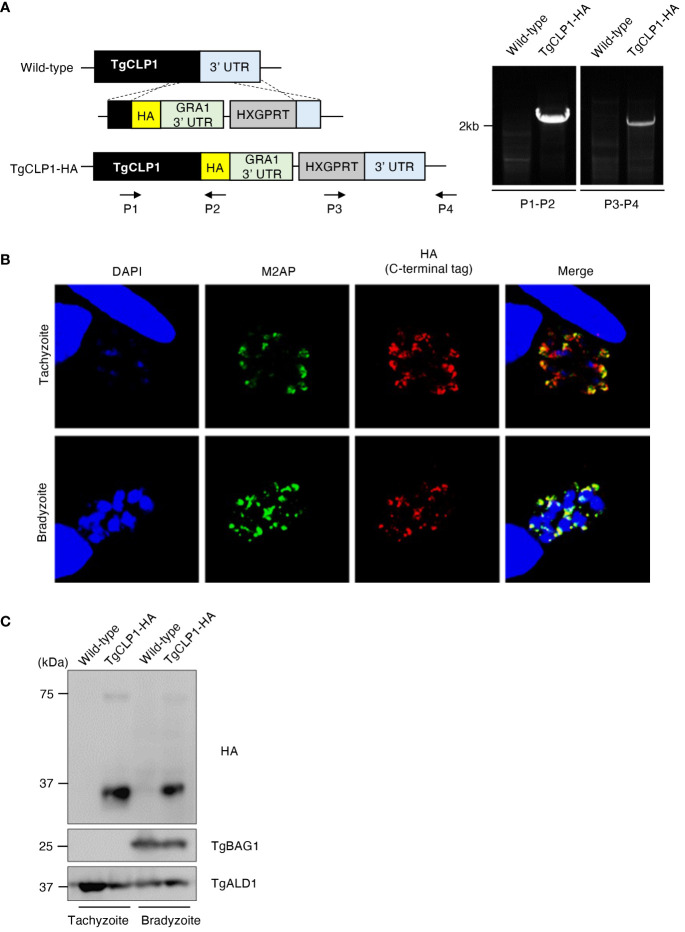
TgCLP1 localizes in the microneme in both the tachyzoite and bradyzoite stages. **(A)** Scheme for generating *T. gondii* with a C-terminal HA-tagged CLP1 using PruΔ*ku80*Δ*hxgprt* strain. The construct for inserting the HA-tag is integrated into the genomic sequence of the TgCLP1 gene through single-homologous recombination. The recombination of the C-terminal HA-tagged TgCLP1 was verified by performing PCR using specific primers (P1-P4) shown in **Supplementary Table S1** and designed for the inserted sequence. **(B, C)** *T. gondii* with a C-terminal HA-tagged TgCLP1 was incubated in normal medium (for tachyzoites) or alkaline medium (for bradyzoites). Then, the localization or expression of TgCLP1 was detected. **(B)** The expression of HA-tagged TgCLP1 was detected by IFA. DAPI, nucleus (blue); M2AP, microneme marker (green); HA, TgCLP1 (red). **(C)** The expression of the CLP1 was detected in the parasite lysates by western blotting. Parasite-specific ALD1 was used as a loading control, and bradyzoite-specific BAG1 was used to show the presence of bradyzoites.

A previous study revealed that C-terminal-tagged TgCLP1 localizes to the microneme in tachyzoites (Huynh and Carruthers, 2009); however, the localization of TgCLP1 in bradyzoites is unknown. To determine the localization of TgCLP1 in bradyzoites, we generated C-terminal HA-tagged TgCLP1 (TgCLP1-HA) parasites (**Figure 2A**). We confirmed that TgCLP1-HA, albeit partially, colocalized with microneme marker protein M2AP in tachyzoites (**Figure 2B** and **Supplementary Figure S1A**), as previously reported (Huynh and Carruthers, 2009). We also found that TgCLP1-HA localized to the microneme in bradyzoites (**Figure 2B** and **Supplementary Figure S1A**). We performed western blot analyses with an anti-HA antibody to confirm the protein expression and molecular weight of HA-tagged TgCLP1 (**Figure 2C**). Interestingly, two band sizes were detected despite the predicted mass of TgCLP1 being approximately 75 kDa (ToxoDB). One was a thick band with a molecular mass of approximately 30 kDa, whereas the other was a thin band with a molecular mass of approximately 75 kDa (**Figure 2C**). To exclude the possibility that the HA tag was expressed with an extra protein, we performed western blotting using anti-TgCLP1 antisera, which identified bands of three sizes: 30 kDa, 45 kDa, and 75 kDa (**Supplementary Figure 3C**), suggesting that although multiple introns exist in *TgCLP1*, the TgClp1 protein is cleaved after translation rather than being alternative splicing. We then performed a CLP1 domain analysis using ToxoDB, CDD/SPARCLE: the conserved domain database in 2020, and SignalP 5.0. We found the predicted signal peptide (residues 1–26) at the N-terminus, and two additional domains: a chitinase domain-like domain (cd00325; residues 130–322: e-value = 4e-19 by the CDD) and a large tegument protein UL36-like domain (PHA03247; residues 321–542: e-value = 4e-04 by the CDD) (**Supplementary Figure S2A**). The observed molecular weight of each fragment obtained by western blotting and the predicted motifs indicated that the cleavage site lies within the C-terminal UL36-like domain, but no definite cleavage site on the domain was detected. The previous study (Huynh and Carruthers, 2009) and our TgCLP1-HA localization findings (**Figure 2B**), may have shown only the processed C-terminal-domain protein.

### Two processed TgCLP1 proteins show different localization patterns in tachyzoites and bradyzoites

**Figure 3:**
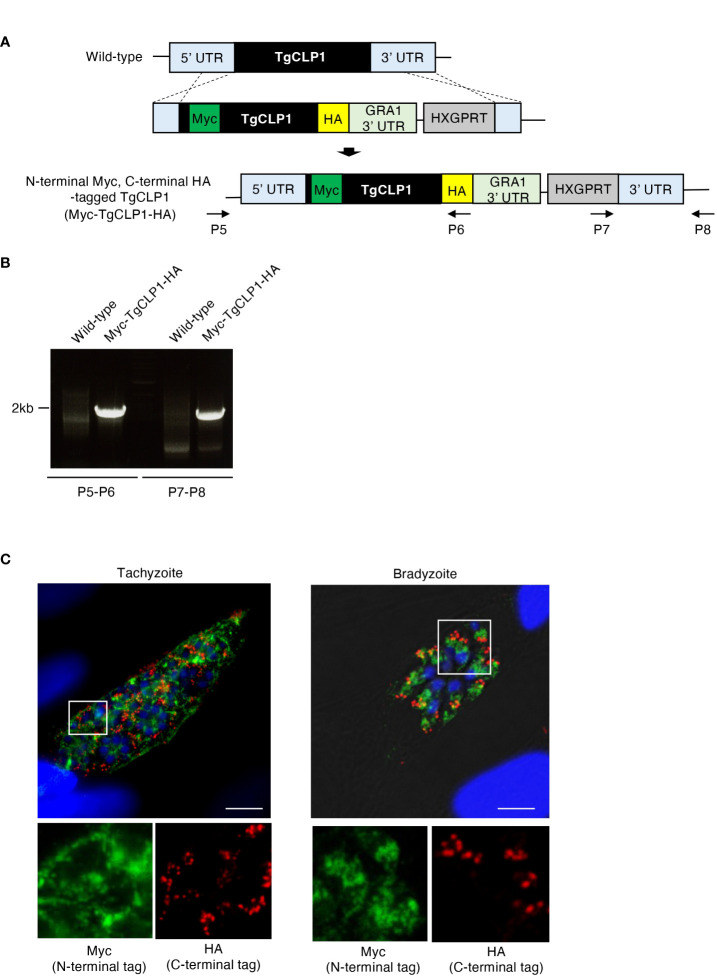
TgCLP1 is not involved in invasion or intracellular replication in the tachyzoite stage. **(A)** Scheme for generating a TgCLP1 knockout *T. gondii* using PruΔ*ku80*Δ*hxgprt* LDH2-GFP strain. The construct for inserting the HXGPRT gene was integrated into the genomic sequence of the TgCLP1 gene through single-homologous recombination. The red bar in TgCLP1 gene represents the region targeted by the sgRNA. The recombination was verified by performing PCR. PCR was performed using specific primers (P9-P14) shown in **Table S1** and designed for the inserted sequence. **(B, C)** HFFs were infected with Wild-type, TgCLP1-KO, or TgCLP1-KO+TgCLP1 *T. gondii*. **(B)** The *T. gondii* infection rate at 3 h post-infection was measured by IFA. **(C)** The *T. gondii* number per vacuole at 24 h post-infection was measured by IFA. Immunofluorescence images are representative of three independent experiments. Indicated values are means ± s.d. (three biological replicates per group from three independent experiments) **(B, C)**. N.S., not significant (Student's t-test).

Since our western blotting suggested that TgCLP1 is cleaved into two fragments of 45 kDa and 30 kDa, we investigate whether each fragment shows different subcellular localization. To determine the localization of the N- or C-terminal-domain-containing TgCLP1 proteins, we generated N-terminal Myc-tagged and C-terminal HA-tagged TgCLP1 (Myc-TgCLP1-HA) parasites (**Figures 3A**, **B**). Then, we performed an IFA to detect the localization of each protein (**Figure 3C**). We found that the N-terminal-domain-containing TgCLP1 protein (Myc-TgCLP1) did not localize with the C-terminal-domain-containing TgCLP1 protein (TgCLP1-HA). Myc-TgCLP1 was found in the cytosol in both tachyzoites and bradyzoites (**Figure 3C**), whereas TgCLP1-HA colocalized with M2AP (**Figure 2B**). These results suggest that TgCLP1 does not exist exclusively within micronemes, in addition, TgCLP1 undergoes cleaving after which the 30-kDa C-terminal fragment containing the large tegument protein UL36-like domain remains in the microneme and the 45-kDa fragment containing the chitinase-like protein is secreted into the cytosol.

### TgCLP1 is not involved in invasion or intracellular replication during the tachyzoite stage

**Figure 4:**
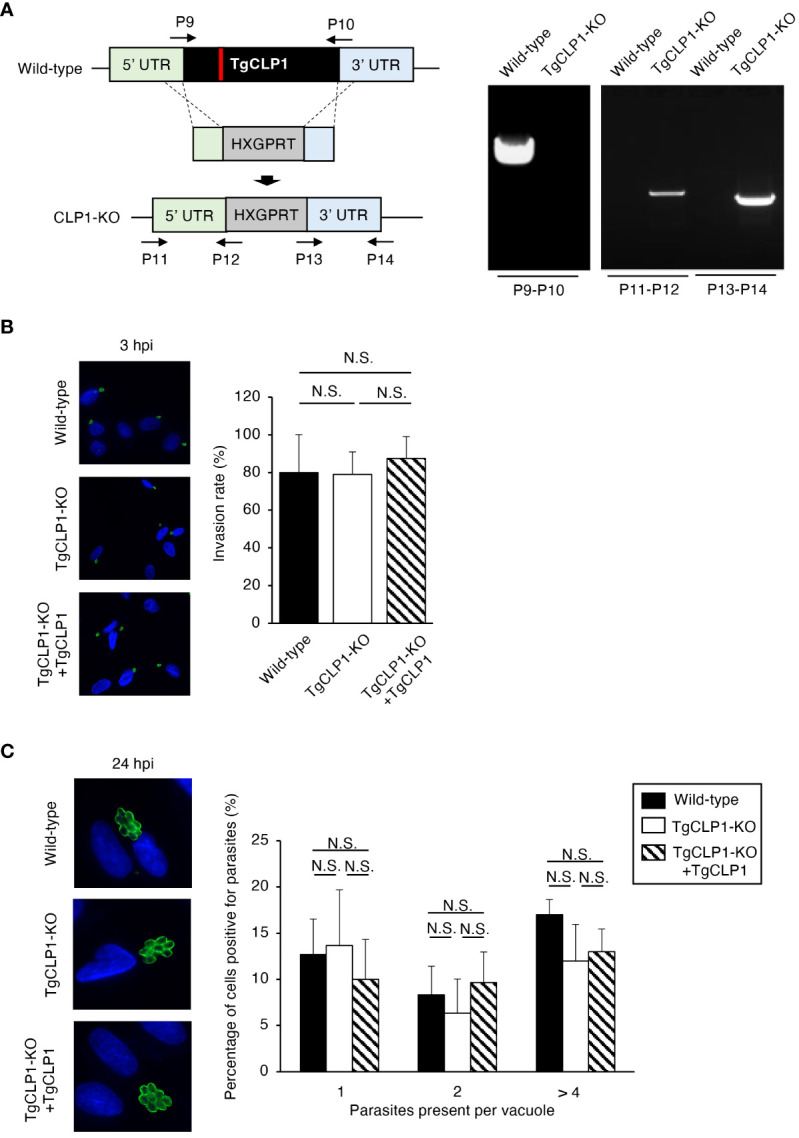
Processed TgCLP1 proteins show different localization. **(A)** Scheme for generating *T. gondii* with N-terminal Myc and C-terminal HA-tagged TgCLP1 using PruΔ*ku80*Δ*hxgprt* strain. The construct for inserting the N-terminal Myc and C-terminal HA-tag was integrated into the genomic sequence of the TgCLP1 gene through single-homologous recombination. **(B)** The recombination was verified by performing PCR using specific primers (P5-P8) shown in **Supplementary Table S1** and designed for the inserted sequence. **(C)** *T. gondii* with N-terminal Myc and C-terminal HA-tagged TgCLP1 was incubated in normal medium (for tachyzoites) or induction medium (for bradyzoites). The expression of Myc-tagged protein or HA-tagged protein was detected by IFA. DAPI, nucleus (blue); Myc, Myc-tagged protein (green); HA, HA-tagged protein (red).

The function of TgCLP1 is unclear in both the tachyzoite and bradyzoite stages. To clarify the role of TgCLP1 during the tachyzoite stage, we generated TgCLP1 knock-out (TgCLP1-KO) parasites (**Figure 4A**) and TgCLP1-KO parasites complemented with wild-type TgCLP1 (TgCLP1-KO+TgCLP1) (**Supplementary Figures S3A**, **B**). The amplification of the coding region of TgCLP1 was not observed in the TgCLP1-KO parasite, and the inserted allele containing HXGPRT was only amplified from the TgCLP1-KO parasite (**Figure 4A**). The deletion of TgCLP1 protein was confirmed by western blotting with anti-TgCLP1 antisera (**Supplementary Figure 3C**). The tachyzoite stage can be divided into three main steps: invasion of the host cell, intracellular replication, and egress from the host cell (Dubey, 2009). First, we tested whether TgCLP1 affected the invasion of *T. gondii* into the host cell. We observed no difference in the numbers of wild-type, TgCLP1-KO, and TgCLP1-KO+TgCLP1 *T. gondii* inside host cells 3 h post-infection (**Figure 4B**), suggesting that TgCLP1 is not involved in parasite invasion. Next, we tested whether TgCLP1 affects the intracellular replication of *T. gondii*. The number of *T. gondii* per vacuoles at 24 h post-infection did not differ among the wild-type, TgCLP1-KO, and TgCLP1-KO+TgCLP1 parasites (**Figure 4C**), which suggests that TgCLP1 is also not involved in intracellular replication.

### TgCLP1 plays a minor role in egress during the tachyzoite stage

**Figure 5:**
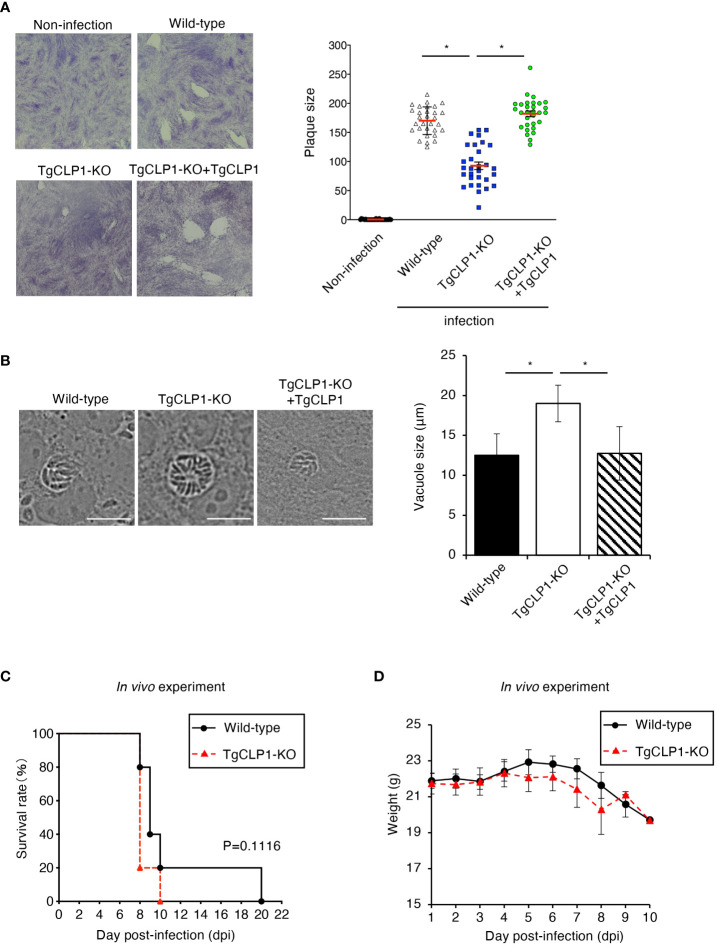
TgCLP1 is somewhat associated with egress during the tachyzoite stage. **(A, B)** HFF cells were infected with Wild-type, TgCLP1-KO, or TgCLP1-KO+TgCLP1 *T. gondii*. **(A)** Plaques were stained at 8 days post-infection. Plaque sizes were measured by using the Image J software. **(B)** The parasites vacuole size was assessed at 3 days post-infection. The parasites vacuole sizes were measured by using the Image J software. Scale bar, 5μm. **(C, D)** WT mice were infected with Wild-type or TgCLP1-KO *T. gondii*. Survival rates **(C)** and weight **(D)** were analyzed. Indicated values are means ± s.d. (three biological replicates per group from three independent experiments) **(A–D)**. *p < 0.05, (Log-rank test or Student’s t-test).

The ability to egress from host cells can be assessed by examining the lytic capacity of host cells (**Figure 5A**). Accordingly, we performed a plaque assay and found that the TgCLP1-KO *T. gondii* produced smaller plaques than the wild-type or TgCLP1-KO+TgCLP1 parasites at 6 days post-infection (**Figure 5A**). This result suggests that TgCLP1 may be linked to egress during the tachyzoite stage. We showed that intracellular replication was not affected by the presence or absence of TgCLP1 (**Figure 4C**). Therefore, we hypothesized that parasitophorous vacuole (PV) size was different between Wild-type and TgCLP1-KO *T. gondii*. To test this hypothesis, we compared the PV size of *T. gondii* with or without TgCLP1 (**Figure 5B**). We found that the PV size of TgCLP1-KO *T. gondii* was larger than that of wild-type or TgCLP1-KO+TgCLP1 parasites (**Figure 5B**). In addition, TgCLP1-KO *T. gondii* spread slowly to host cells (**Supplementary Figure S4A**), supporting our finding on the effect of TgCLP1 deficiency on egress and PV size (**Figures 5A**, **B**). Finally, we examined the influence of TgCLP1 on *T. gondii* infection in mice using an *in vivo* acute infection model (**Figures 5C, D**). We found no difference in the survival (**Figure 5C**) or weight loss (**Figure 5D**) of the mice infected with *T. gondii* regardless of TgCLP1 expression. These results indicate that, although TgCLP1 is involved in egress during the tachyzoite stage, its deficiency results in a moderate phenotype and does not affect acute parasite infection *in vivo*.

### TgCLP1 is not involved in cyst formation in the bradyzoite stage

**Figure 6:**
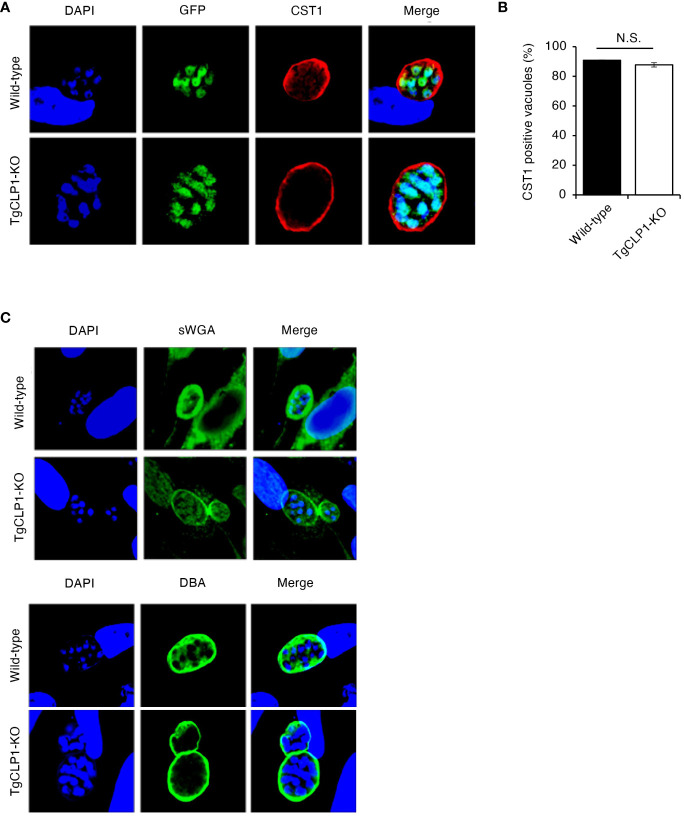
TgCLP1 is not involve in cyst formation in the bradyzoite stage. **(A–C)** HFF cells were infected with Wild-type or TgCLP1-KO *T. gondii*. Parasites treated with induction medium for 4 days were analyzed by IFA. **(A)** The expression of CST1 was detected by IFA. DAPI, nucleus (blue); GFP, bradyzoite marker (green); CST1, the cyst wall marker (red). **(B)** The ratio of CST1-positive vacuoles was counted. **(C)** The morphology of the cyst wall was analyzed by using sWGA-FITC or DBA-FITC. Indicated values are means ± s.d. (three biological replicates per group from three independent experiments) **(B)** N.S., not significant (Student’s *t*-test).

To clarify the contribution of TgCLP1 during the bradyzoite stage, we assessed whether TgCLP1 expression is important for tachyzoite to bradyzoite conversion. *T. gondii* tachyzoites were cultured under alkaline stress conditions, and stage conversion was confirmed by staining of the cyst wall glycoprotein CST1, a bradyzoite-specific marker (Zhang et al., 2001). First, we compared the stage conversion rates of wild-type and TgCLP1-KO *T. gondii* by counting CST1-positive vacuoles (**Figures 6A, B**). We found that the ratio of CST1-positive vacuoles was comparable between wild-type and TgCLP1-KO parasites (**Figure 6B**). Furthermore, to confirm whether cyst wall formation was normal in the absence of TgCLP1, we investigated other cyst wall components by using sWGA and DBA, which bind to cyst wall lectins (**Figure 6C**). Both sWGA and DBA showed normal staining in wild-type and TgCLP1-KO parasites (**Figure 6C**). These results suggest that TgCLP1 is not involved in cyst formation in the bradyzoite stage.

### TgCLP1 is involved in bradyzoite reactivation

**Figure 7:**
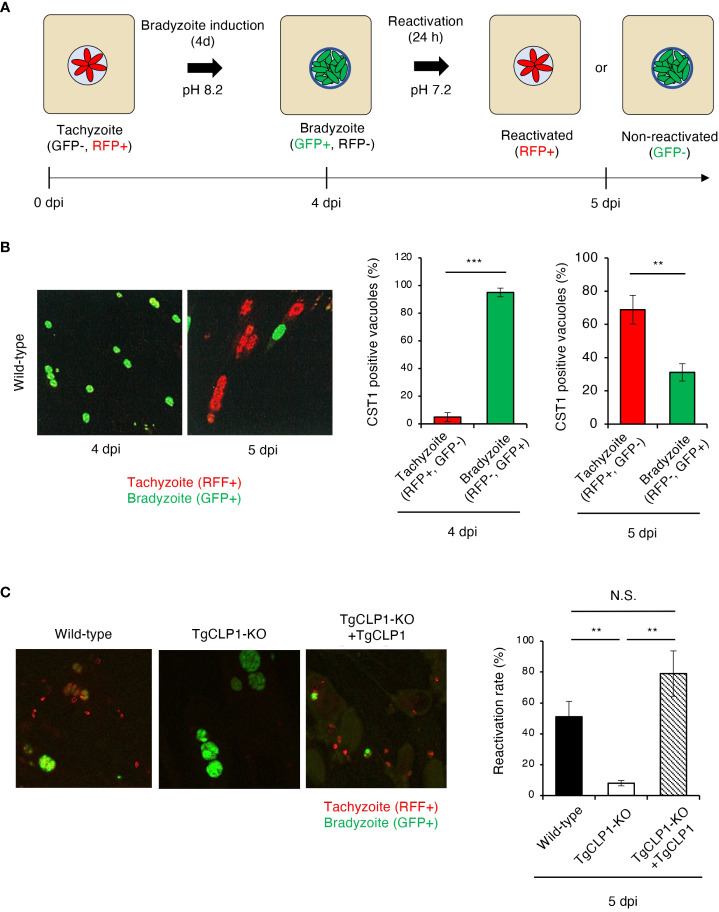
TgCLP1 is required for bradyzoite reactivation. **(A, B)** Establishment of a reactivation assay. HFF cells were infected with *T. gondii* PruΔ*ku80*Δ*hxgprt* LDH2-GFP. Parasites were treated with induction medium for 4 days post-infection (dpi). Then, *T. gondii* was incubated in normal medium for 1 day. RFP-positive parasites are defined as reactivated parasites. GFP-positive parasites are defined as non-reactivated parasites. **(A)** Images illustrating the reactivation assay. **(B)** Representative IFA images at 4 or 5 dpi (left). The ratio of RFP- or GFP-positive parasites per CST1-positive vacuole was counted by IFA. **(C)** HFF cells were infected with Wild-type, TgCLP1-KO, or TgCLP1-KO+TgCLP1 *T. gondii*, and incubated in induction medium for 4 days. Then, *T. gondii* was incubated in normal medium for 24 h The ratio of reactivated *T. gondii* after reactivation induction was calculated by IFA (GFP-positive parasites per CST1-positive vacuole). Indicated values are means ± s.d. (three biological replicates per group from three independent experiments) **(B, C)**. **p < 0.01, ***p < 0.001; N.S., not significant; (Student’s t-test).

*In vitro* bradyzoite reactivation for *T. gondii* has rarely been studied; therefore, in the present study, we established an assay to assess it (**Figure 7A**). Using our method, we confirmed that more than 90% of wild-type *T. gondii* differentiated into bradyzoites when cultured under induction conditions, and more than 60% of bradyzoites reactivated after reactivation stimulation (**Figure 7B**). Then, we first tested localization of TgCLP1 in each stage by using Myc-CLP1-HA *T. gondii* using this method (**Supplementary Figure S5**). During the early to mid-tachyzoite stages, Myc-tagged CLP1 is present around the apical complex and on the surface of the parasite, while towards the end of egress from host cells, it is also located around the host cell membrane (**Supplementary Figure S5A**). On the other hand, during the bradyzoite stage, Myc-tagged CLP1 was only found around the apical complex or surface of parasite, but after reactivation stimulation, it also relocated to reside near the cyst wall (**Supplementary Figure S5B**). Additionally, HA-tagged CLP1 was found to be present at the apical complex throughout all stages (**Supplementary Figures S5A, B**). These results supported our finding about C-terminal CLP1 cleavage, and suggested the present of N-terminal CLP1 contains a signal peptide (**Figure 3B** and **Supplementary Figure S2A**). And also, these results suggested that CLP1 has some interaction with the cyst wall during reactivation. Then, we next tested whether TgCLP1 has an important role in reactivation from bradyzoite to tachyzoite (**Figure 7C**). After reactivation stimulation, approximately 50% of wild-type *T. gondii* and 80% of TgCLP1-KO+TgCLP1 *T. gondii* were reactivated (**Figure 7C**). In contrast, less than 10% of TgCLP1-KO *T. gondii* were reactivated (**Figure 7C**). Taken together, these results indicate that TgCLP1 has an important role in bradyzoite to tachyzoite conversion.

## Discussion

In the present study, we characterized *T. gondii* CLP1 (TGME49_293770). First, we showed that TgCLP1 is expressed during both the tachyzoite and bradyzoite stage. Then, we performed phenotypic analyses using TgCLP1-KO *T. gondii* and found that TgCLP1 plays an important role in bradyzoite reactivation.

*T. gondii* has unique life cycles in its tachyzoite and bradyzoite stages in intermediate hosts (Dubey, 2009). Various stage-specific molecules, such as the parasite surface antigen SAG1, which is expressed specifically in the tachyzoite stage (Opitz et al., 2002) and the bradyzoite antigen BAG1, which is expressed specifically in the bradyzoite stage (Tomavo et al., 1991), have long been investigated. However, the functions of the small phenotypic genes expressed throughout the life cycle of *T. gondii* have remained largely unknown. In this study, TgCLP1 was found to be expressed during both the tachyzoite and bradyzoite stages. We successfully generated TgCLP1-KO *T. gondii*, indicating that TgCLP1 is not essential in the tachyzoite stage. We could not find any effect of TgCLP1 on host cell invasion or intracellular replication; a slight effect on egress was found, however there was no effect on *in vivo* acute infection. Furthermore, we found no effect of TgCLP1 on stage conversion from tachyzoite to bradyzoite or on cyst wall formation. Therefore, TgCLP1 may not be a promising drug target for the acute phase of infection or for the prevention of latent infection.

Although TgCLP1-KO *T. gondii* did not show any phenotype in stage conversion from tachyzoite to bradyzoite, interestingly, we found that TgCLP1 deficiency leads to significant inhibition of bradyzoite reactivation. Few studies on cyst burden have been conducted, and the molecular mechanism of bradyzoite reactivation is completely unknown. In this study, we succeeded in finding this role of TgCLP1 by establishing an experimental system to evaluate bradyzoite reactivation. Despite many people being at risk of reactivation of latent *T. gondii* cysts, there is currently no curative treatment available. Therefore, our discovery that TgCLP1 regulates cyst reactivation represents a conceptual advance in our understanding of how to control latent *T. gondii* infection to prevent reactivation. In addition, the novel experimental system we have established to evaluate bradyzoite reactivation could be used to identify new drug targets in the future.

The wall of tissue cysts is formed from PVs. This wall can be stained with periodic acid–Schiff, and the cyst wall lectins can bind DBA and sWGA. These properties indicate that the cyst wall contains polysaccharides, such as *N*-acetylgalactosamine, which binds DBA, and *N*-acetylglucosamine, which binds sWGA. The cyst wall can be digested with chitinase because it contains a chitin β-1,4-linked polymer of *N*-acetylglucosamine (Boothroyd et al., 1997; Weiss and Kim, 2000). A previous study showed that, upon disruption of the predicted transporter of polysaccharides (nucleotide-sugar transporter TgNST1), the cyst wall cannot be stained with lectins due to the absence of glycosylation (Caffaro et al., 2013). In addition, cyst formation *in vivo* can be inhibited by a lack of TgNST1 (Caffaro et al., 2013). These findings suggest that the polysaccharides in the cyst wall play important roles in sustaining latent infection.

In the present study, we showed that a chitinase-like protein is required for bradyzoite reactivation, which suggests that the parasite uses a chitinase-like protein to escape from cysts as part of its virulence mechanism. Although several studies have identified triggers of cyst formation and key factors to maintain the latent infection, there is little information about bradyzoite reactivation. *In vitro*-generated bradyzoites revert to tachyzoites upon the removal of the stress agents used to induce bradyzoites, suggesting that cellular stress is necessary to maintain cyst formation (Sullivan and Jeffers, 2012). It has also been reported that latently infected parasites are reactivated when the host immune system is suppressed with dexamethasone in *in vivo* models (Nicoll et al., 1997; Dellacasa-Lindberg et al., 2007). In contrast, TgCLP1 which we characterized here, is the first reported intrinsic *Toxoplasma* trigger of bradyzoite to tachyzoite differentiation.

Previous work has demonstrated that the cyst wall can be digested by exogenous chitinase, suggesting that *T. gondii* might synthesize the *N*-acetylglucosamine polymer and transport it to the cyst wall (Boothroyd et al., 1997). Other studies have suggested that high expression of host chitinase can reduce the cyst formation in the brain because the cyst wall, which may include chitin, was digested (Nance et al., 2012). In this study we asked whether endogenous TgCLP1 is required for the *T. gondii* life cycle, and how TgCLP1 is used by *T. gondii*. We found that TgCLP1-KO *T. gondii* only exhibited an obvious phenotype in bradyzoite reactivation. This finding provides support for the presence of chitin in the cyst wall, although the *T. gondii* chitin synthetase has not yet been characterized. In addition, our finding suggests that TgCLP1 may digest cyst wall chitin when the bradyzoite-inducing stress is removed, facilitating parasite conversion from bradyzoite to tachyzoite, rapid division, egress from the PV, and transmission to other cells. Further work is required to determine the direct interaction between chitin in the cyst wall and TgCLP1.

Based on our analysis using NCBI’s Conserved Domain Database (CDD) and SignalP, it was predicted that TgCLP1 possesses an N-terminal signal sequence and a kinase domain in the region from the central part to the N-terminus of the TgCLP1 protein (**Supplementary Figure S1A**). In addition, western blot analysis using anti-TgCLP1 antibodies revealed that TgCLP1 is cleaved into two fragments: a 45-kDa N-terminal fragment containing the kinase domain and a 30-kDa C-terminal fragment (**Figure 4C**). Furthermore, immunofluorescence analysis using an IFA against the Myc-tagged N-terminal protein (chitinase domain-like) showed that the 45-kDa fragment localizes to the cytoplasm of the parasite, whereas an IFA against the HA-tagged C-terminal protein (large tegument protein UL36-like) revealed that the 30-kDa fragment localizes to the micronemes (**Figure 3C**). Taken together, our findings indicate that translated TgCLP1 undergoes translocation to the micronemes through the signal sequence at the N-terminus and is subsequently cleaved into the 45-kDa and 30-kDa fragments. Then, the 45-kDa fragment containing the kinase domain relocalizes to the cytoplasm. If TgCLP1 does digest the cyst wall, that would suggest that it should be secreted into the PV. We demonstrated that TgCLP1 undergoes cleaving, and the predicted active site of the chitinase in TgCLP1 is retained in the N-terminal half of the TgCLP1 sequence. Therefore, the localization of the N-terminus of TgCLP1 should also be determined in the future to evaluate whether TgCLP1 is secreted upon recrudescence.

In summary, we revealed that TgCLP1 is involved in *T. gondii* conversion from bradyzoites to tachyzoites. Our results suggest that TgCLP1 may be a key factor in recrudescence in immunosuppressed individuals. Moreover, our findings provide insight into the mechanisms of latent infection and reactivation, with implications for the development of strategies to treat toxoplasmosis in immunocompromised individuals.

## Data availability statement

The datasets presented in this study can be found in online repositories. The names of the repository/repositories and accession number(s) can be found in the article/**Supplementary Material**.

## Ethics statement

The animal study was approved by the Research Ethics Review Committee of the Obihiro University of Agriculture and Veterinary Medicine (approval numbers 29-56). The study was conducted in accordance with the local legislation and institutional requirements.

## Author contributions

HB: Writing – original draft, Writing – review & editing. YM: Writing – original draft, Writing – review & editing. YH: Writing – original draft, Writing – review & editing. TS: Data curation, Formal analysis, Resources, Visualization, Writing – review & editing. YF: Formal analysis, Methodology, Resources, Visualization, Writing – review & editing. DB: Methodology, Resources, Supervision, Visualization, Writing – review & editing. BF: Methodology, Resources, Supervision, Visualization, Writing – review & editing. KK: Writing – original draft, Writing – review & editing.

## Supplementary material

Supplementary Figure 1 Images of parasitophorous vacuole. **(A)** Representative images of parasitophorous vacuole in tachyzoite or bradyzoite stage.

Supplementary Figure 2 Sequence analysis of TgCLP1. **(A)** Organization of the TgCLP1 sequence. CLP1 domain analyses were performed by ToxoDB, CDD/SPARCLE, and SignalP 5.0. Blue: Signal peptide; residues 1–26. Red: Chitinase domain-like (cd00325); residues 130–322, e-value = 4e-19. Green: large tegument protein UL36-like (PHA03247); residues 321–542, e-value = 4e-04. E-values were calculated by CDD.

Supplementary Figure 3 Complementation of TgCLP1 knockout parasites. **(A)** Scheme for generating a TgCLP1 complement in TgCLP1 knockout *T. gondii* using PruΔ*ku80*Δ*hxgprt* LDH2-GFP strain. The construct for inserting the wild-type TgCLP1 gene was placed into the genomic sequence of the UPRT gene through single-homologous recombination. The red bar in UPRT gene represents the region targeted by the sgRNA. **(B)** The insertion of the wild-type TgCLP1 with the HA tag was verified by PCR with specific primers (P15-P18) of inserting sequence. The complementation was verified by performing a PCR using specific primers (P15-P18) shown in **Table S1** and designed for the inserted sequence. **(C)** Scheme for generating a TgCLP1 knockout *T. gondii* using PruΔ*ku80*Δ*hxgprt* LDH2-GFP strain. The construct for inserting the HXGPRT gene was integrated into the genomic sequence of the TgCLP1 gene through single-homologous recombination. The red bar in TgCLP1 gene represents the region targeted by the sgRNA. The recombination was verified by western blotting. The expression of TgCLP1 was detected in the parasite lysates by western blotting. Parasite-specific ALD1 was used as a loading control. TgCLP1-KO, TgCLP1 knockout *T. gondii*; TgCLP1-KO+TgCLP1, wild-type TgCLP1 complemented.

Supplementary Figure 4 Observation of parasite egress. **(A)** HFF cells were infected with Wild-type, TgCLP1-KO, or TgCLP1-KO+TgCLP1 *T. gondii*. At 3 days post-infection, parasite expansion was observed by use of fluorescence microscopy. Representative images are provided in the figure. Hoechst, nucleus (blue); RFP, tachyzoite (green).

Supplementary Figure 5 Time-resolved localization of TgCLP1. **(A, B)** *T. gondii* with N-terminal Myc and C-terminal HA-tagged TgCLP1 was incubated in normal medium (for tachyzoites) or induction medium (for bradyzoites). The expression of Myc-tagged protein or HA-tagged protein was detected by IFA. DAPI, nucleus (blue); Myc, Myc-tagged protein (green); HA, HA-tagged protein (red). **(A)** At 24 or 72 hours post-infection in the normal medium, tachyzoite stage parasites were observed. **(B)** At 4 days post-infection in the induction medium, bradyzoite stage non-activated parasites or activated parasites (12 hours reactivation) were observed.

## References

[B1] Alaganan A. Fentress S. J. Tang K. Wang Q. Sibley L. D. (2014). Toxoplasma GRA7 effector increases turnover of immunity-related GTPases and contributes to acute virulence in the mouse. Proc. Natl. Acad. Sci. U.S.A. 111, 1126–1131. doi: 10.1073/pnas.1313501111 24390541 PMC3903209

[B2] Alday P. H. Doggett J. S. (2017). Drugs in development for toxoplasmosis: advances, challenges, and current status. Drug Des. Devel. Ther. 11, 273–293. doi: 10.2147/dddt.S60973 PMC5279849 28182168

[B3] Bando H. Fukuda Y. Watanabe N. Olawale J. T. Kato K. (2021). Depletion of intracellular glutamine pools triggers toxoplasma gondii stage conversion in human glutamatergic neurons. Front. Cell Infect. Microbiol. 11. doi: 10.3389/fcimb.2021.788303 PMC8793678 35096641

[B4] Bando H. Lee Y. Sakaguchi N. Pradipta A. Ma J. S. Tanaka S.. (2018b). Inducible nitric oxide synthase is a key host factor for toxoplasma GRA15-dependent disruption of the gamma interferon-induced antiparasitic human response. MBio 9, e01738-18. doi: 10.1128/mBio.01738-18 PMC6178625 30301855

[B5] Bando H. Lee Y. Sakaguchi N. Pradipta A. Sakamoto R. Tanaka S.. (2019). Toxoplasma effector GRA15-dependent suppression of IFN-γ-induced antiparasitic response in human neurons. Front. Cell Infect. Microbiol. 9. doi: 10.3389/fcimb.2019.00140 PMC6504700 31119110

[B6] Bando H. Sakaguchi N. Lee Y. Pradipta A. Ma J. S. Tanaka S.. (2018a). Toxoplasma effector tgIST targets host IDO1 to antagonize the IFN-gamma-induced anti-parasitic response in human cells. Front. Immunol. 9. doi: 10.3389/fimmu.2018.02073 PMC6156249 30283439

[B7] Batz M. B. Hoffmann S. Morris J. G. J. (2012). Ranking the disease burden of 14 pathogens in food sources in the United States using attribution data from outbreak investigations and expert elicitation. J. Food Prot. 75, 1278–1291. doi: 10.4315/0362-028X.JFP-11-418 22980012

[B8] Behnke M. S. Khan A. Wootton J. C. Dubey J. P. Tang K. Sibley L. D. (2011). Virulence differences in Toxoplasma mediated by amplification of a family of polymorphic pseudokinases. Proc. Natl. Acad. Sci. U.S.A. 108, 9631–9636. doi: 10.1073/pnas.1015338108 21586633 PMC3111276

[B9] Boothroyd J. C. (2009). Toxoplasma gondii: 25 years and 25 major advances for the field. Int. J. Parasitol. 39, 935–946. doi: 10.1016/j.ijpara.2009.02.003 19630140 PMC2895946

[B10] Boothroyd J. C. Black M. Bonnefoy S. Hehl A. Knoll L. J. Manger I. D.. (1997). Genetic and biochemical analysis of development in Toxoplasma gondii. Philos. Trans. R. Soc. Lond. B. Biol. Sci. 352, 1347–1354. doi: 10.1098/rstb.1997.0119 9355126 PMC1692023

[B11] Buchholz K. R. Fritz H. M. Chen X. Durbin-Johnson B. Rocke D. M. Ferguson D. J.. (2011). Identification of tissue cyst wall components by transcriptome analysis of *in vivo* and *in vitro Toxoplasma gondii* bradyzoites. Eukaryot. Cell 10, 1637–1647. doi: 10.1128/ec.05182-11 22021236 PMC3232729

[B12] Caffaro C. E. Koshy A. A. Liu L. Zeiner G. M. Hirschberg C. B. Boothroyd J. C. (2013). A nucleotide sugar transporter involved in glycosylation of the Toxoplasma tissue cyst wall is required for efficient persistence of bradyzoites. PloS Pathog. 9, e1003331. doi: 10.1371/journal.ppat.1003331 23658519 PMC3642066

[B13] Cérède O. Dubremetz J. F. Bout D. Lebrun M. (2002). The Toxoplasma gondii protein MIC3 requires pro-peptide cleavage and dimerization to function as adhesin. EMBO J. 21, 2526–2536. doi: 10.1093/emboj/21.11.2526 12032066 PMC126022

[B14] Cheng J. H. Xu X. Li Y. B. Zhao X. D. Aosai F. Shi S. Y.. (2020). Arctigenin ameliorates depression-like behaviors in Toxoplasma gondii-infected intermediate hosts via the TLR4/NF-κB and TNFR1/NF-κB signaling pathways. Int. Immunopharmacol. 82, 106302. doi: 10.1016/j.intimp.2020.106302 32086097

[B15] Costa Mendonça-Natividade F. Ricci-Azevedo R. De Oliveira Thomaz S. M. Roque-Barreira M. C. (2020b). Production and characterization of MIC1: A lectin from toxoplasma gondii. Methods Mol. Biol. 2132, 391–400. doi: 10.1007/978-1-0716-0430-4_38 32306346

[B16] Costa Mendonça-Natividade F. Ricci-Azevedo R. Roque-Barreira M. C. (2020a). MIC4 from toxoplasma gondii: A lectin acting as a toll-like receptor agonist. Methods Mol. Biol. 2132, 379–389. doi: 10.1007/978-1-0716-0430-4_37 32306345

[B17] Dellacasa-Lindberg I. Hitziger N. Barragan A. (2007). Localized recrudescence of Toxoplasma infections in the central nervous system of immunocompromised mice assessed by *in vivo* bioluminescence imaging. Microbes Infect. 9, 1291–1298. doi: 10.1016/j.micinf.2007.06.003 17897859

[B18] Dubey J. P. (2009). History of the discovery of the life cycle of Toxoplasma gondii. Int. J. Parasitol. 39, 877–882. doi: 10.1016/j.ijpara.2009.01.005 19630138

[B19] Dubey J. P. (2010). Toxoplasmosis of Animals and Humans (Boca Raton: CRC Press). doi: 10.1201/9781420092370

[B20] El-On J. Peiser J. (2003). [Toxoplasma and toxoplasmosis]. Harefuah 142, 48–55, 77. 12647490

[B21] Etheridge R. D. Alaganan A. Tang K. Lou H. J. Turk B. E. Sibley L. D. (2014). The Toxoplasma pseudokinase ROP5 forms complexes with ROP18 and ROP17 kinases that synergize to control acute virulence in mice. Cell Host Microbe 15, 537–550. doi: 10.1016/j.chom.2014.04.002 24832449 PMC4086214

[B22] Fentress S. J. Behnke M. S. Dunay I. R. Mashayekhi M. Rommereim L. M. Fox B. A.. (2010). Phosphorylation of immunity-related GTPases by a Toxoplasma gondii-secreted kinase promotes macrophage survival and virulence. Cell Host Microbe 8, 484–495. doi: 10.1016/j.chom.2010.11.005 21147463 PMC3013631

[B23] Ferguson D. J. Huskinson-Mark J. Araujo F. G. Remington J. S. (1994). An ultrastructural study of the effect of treatment with atovaquone in brains of mice chronically infected with the ME49 strain of Toxoplasma gondii. Int. J. Exp. Pathol. 75, 111–116. 8199003 PMC2002108

[B24] Fox B. A. Falla A. Rommereim L. M. Tomita T. Gigley J. P. Mercier C.. (2011). Type II Toxoplasma gondii KU80 knockout strains enable functional analysis of genes required for cyst development and latent infection. Eukaryot. Cell 10, 1193–1206. doi: 10.1128/ec.00297-10 21531875 PMC3187049

[B25] Frenkel J. K. Remington J. S. (1980). Hepatitis in toxoplasmosis. N. Engl. J. Med. 302, 178–179. 7350455

[B26] Gay G. Braun L. Brenier-Pinchart M. P. Vollaire J. Josserand V. Bertini R. L.. (2016). Toxoplasma gondii TgIST co-opts host chromatin repressors dampening STAT1-dependent gene regulation and IFN-gamma-mediated host defenses. J. Exp. Med. 213, 1779–1798. doi: 10.1084/jem.20160340 27503074 PMC4995087

[B27] Gazzinelli R. T. Mendonca-Neto R. Lilue J. Howard J. Sher A. (2014). Innate resistance against Toxoplasma gondii: an evolutionary tale of mice, cats, and men. Cell Host Microbe 15, 132–138. doi: 10.1016/j.chom.2014.01.004 24528860 PMC4006104

[B28] Gorfu G. Cirelli K. M. Melo M. B. Mayer-Barber K. Crown D. Koller B. H.. (2014). Dual role for inflammasome sensors NLRP1 and NLRP3 in murine resistance to Toxoplasma gondii. MBio 5, e01117-13. doi: 10.1128/mBio.01117-13 PMC3944820 24549849

[B29] Gormley P. D. Pavesio C. E. Minnasian D. Lightman S. (1998). Effects of drug therapy on Toxoplasma cysts in an animal model of acute and chronic disease. Invest. Ophthalmol. Vis. Sci. 39, 1171–1175. 9620076

[B30] Gov L. Karimzadeh A. Ueno N. Lodoen M. B. (2013). Human innate immunity to Toxoplasma gondii is mediated by host caspase-1 and ASC and parasite GRA15. MBio 4, e00255-13. doi: 10.1128/mBio.00255-13 PMC3705447 23839215

[B31] Guevara R. B. Fox B. A. Bzik D. J. (2021). A family of toxoplasma gondii genes related to GRA12 regulate cyst burdens and cyst reactivation. mSphere 6, e00182-21. doi: 10.1128/mSphere.00182-21 PMC8546695 33883265

[B32] Hakimi M. A. Olias P. Sibley L. D. (2017). Toxoplasma effectors targeting host signaling and transcription. Clin. Microbiol. Rev. 30, 615–645. doi: 10.1128/cmr.00005-17 28404792 PMC5475222

[B33] Hunter C. A. Sibley L. D. (2012). Modulation of innate immunity by Toxoplasma gondii virulence effectors. Nat. Rev. Microbiol. 10, 766–778. doi: 10.1038/nrmicro2858 23070557 PMC3689224

[B34] Huynh M. H. Carruthers V. B. (2009). Tagging of endogenous genes in a Toxoplasma gondii strain lacking Ku80. Eukaryot. Cell 8, 530–539. doi: 10.1128/ec.00358-08 19218426 PMC2669203

[B35] Jensen K. D. Hu K. Whitmarsh R. J. Hassan M. A. Julien L. Lu D.. (2013). Toxoplasma gondii rhoptry 16 kinase promotes host resistance to oral infection and intestinal inflammation only in the context of the dense granule protein GRA15. Infect. Immun. 81, 2156–2167. doi: 10.1128/IAI.01185-12 23545295 PMC3676013

[B36] Loeches Yagüe B. Rico-Nieto A. Refoyo Salicio E. Iniesta Manjavacas Á,M. (2023). Myocarditis by Toxoplasma gondii in an immunocompetent young man. Enferm. Infecc. Microbiol. Clin. (Engl. Ed). 41, 375–376. doi: 10.1016/j.eimce.2023.01.004 36670041

[B37] Macmicking J. D. (2012). Interferon-inducible effector mechanisms in cell-autonomous immunity. Nat. Rev. Immunol. 12, 367–382. doi: 10.1038/nri3210 22531325 PMC4150610

[B38] Montoya J. G. Remington J. S. (2008). Management of Toxoplasma gondii infection during pregnancy. Clin. Infect. Dis. 47, 554–566. doi: 10.1086/590149 18624630

[B39] Nance J. P. Vannella K. M. Worth D. David C. Carter D. Noor S.. (2012). Chitinase dependent control of protozoan cyst burden in the brain. PloS Pathog. 8, e1002990. doi: 10.1371/journal.ppat.1002990 23209401 PMC3510238

[B40] Nicoll S. Wright S. Maley S. W. Burns S. Buxton D. (1997). A mouse model of recrudescence of Toxoplasma gondii infection. J. Med. Microbiol. 46, 263–266. doi: 10.1099/00222615-46-3-263 9126829

[B41] Olias P. Etheridge R. D. Zhang Y. Holtzman M. J. Sibley L. D. (2016). Toxoplasma effector recruits the mi-2/nuRD complex to repress STAT1 transcription and block IFN-γ-dependent gene expression. Cell Host Microbe 20, 72–82. doi: 10.1016/j.chom.2016.06.006 27414498 PMC4947229

[B42] Opitz C. Di Cristina M. Reiss M. Ruppert T. Crisanti A. Soldati D. (2002). Intramembrane cleavage of microneme proteins at the surface of the apicomplexan parasite Toxoplasma gondii. EMBO J. 21, 1577–1585. doi: 10.1093/emboj/21.7.1577 11927542 PMC125952

[B43] Pappas G. Roussos N. Falagas M. E. (2009). Toxoplasmosis snapshots: global status of Toxoplasma gondii seroprevalence and implications for pregnancy and congenital toxoplasmosis. Int. J. Parasitol. 39, 1385–1394. doi: 10.1016/j.ijpara.2009.04.003 19433092

[B44] Reese M. L. Zeiner G. M. Saeij J. P. Boothroyd J. C. Boyle J. P. (2011). Polymorphic family of injected pseudokinases is paramount in Toxoplasma virulence. Proc. Natl. Acad. Sci. U.S.A. 108, 9625–9630. doi: 10.1073/pnas.1015980108 21436047 PMC3111280

[B45] Robert-Gangneux F. Darde M. L. (2012). Epidemiology of and diagnostic strategies for toxoplasmosis. Clin. Microbiol. Rev. 25, 264–296. doi: 10.1128/cmr.05013-11 22491772 PMC3346298

[B46] Roozbehani M. Falak R. Mohammadi M. Hemphill A. Razmjou E. Meamar A. R.. (2018). Characterization of a multi-epitope peptide with selective MHC-binding capabilities encapsulated in PLGA nanoparticles as a novel vaccine candidate against Toxoplasma gondii infection. Vaccine 36, 6124–6132. doi: 10.1016/j.vaccine.2018.08.068 30181047

[B47] Rosowski E. E. Nguyen Q. P. Camejo A. Spooner E. Saeij J. P. (2014). Toxoplasma gondii Inhibits gamma interferon (IFN-gamma)- and IFN-beta-induced host cell STAT1 transcriptional activity by increasing the association of STAT1 with DNA. Infect. Immun. 82, 706–719. doi: 10.1128/iai.01291-13 24478085 PMC3911376

[B48] Rosowski E. E. Saeij J. P. (2012). Toxoplasma gondii clonal strains all inhibit STAT1 transcriptional activity but polymorphic effectors differentially modulate IFNgamma induced gene expression and STAT1 phosphorylation. PloS One 7, e51448. doi: 10.1371/journal.pone.0051448 23240025 PMC3519884

[B49] Santos J. M. Ferguson D. J. Blackman M. J. Soldati-Favre D. (2011). Intramembrane cleavage of AMA1 triggers Toxoplasma to switch from an invasive to a replicative mode. Science 331, 473–477. doi: 10.1126/science.1199284 21205639

[B50] Schlüter D. Barragan A. (2019). Advances and challenges in understanding cerebral toxoplasmosis. Front. Immunol. 10. doi: 10.3389/fimmu.2019.00242 PMC6401564 30873157

[B51] Schwarz J. A. Fouts A. E. Cummings C. A. Ferguson D. J. Boothroyd J. C. (2005). A novel rhoptry protein in Toxoplasma gondii bradyzoites and merozoites. Mol. Biochem. Parasitol. 144, 159–166. doi: 10.1016/j.molbiopara.2005.08.011 16182390

[B52] Steinfeldt T. Konen-Waisman S. Tong L. Pawlowski N. Lamkemeyer T. Sibley L. D.. (2010). Phosphorylation of mouse immunity-related GTPase (IRG) resistance proteins is an evasion strategy for virulent Toxoplasma gondii. PloS Biol. 8, e1000576. doi: 10.1371/journal.pbio.1000576 21203588 PMC3006384

[B53] Sullivan W. J. Jr. Jeffers V. (2012). Mechanisms of Toxoplasma gondii persistence and latency. FEMS Microbiol. Rev. 36, 717–733. doi: 10.1111/j.1574-6976.2011.00305.x 22091606 PMC3319474

[B54] Sutterland A. L. Fond G. Kuin A. Koeter M. W. Lutter R. Van Gool T.. (2015). Beyond the association. Toxoplasma gondii in schizophrenia, bipolar disorder, and addiction: systematic review and meta-analysis. Acta Psychiatr. Scand. 132, 161–179. doi: 10.1111/acps.12423 25877655

[B55] Tomavo S. Fortier B. Soete M. Ansel C. Camus D. Dubremetz J. F. (1991). Characterization of bradyzoite-specific antigens of Toxoplasma gondii. Infect. Immun. 59, 3750–3753. doi: 10.1128/iai.59.10.3750-3753.1991 1894373 PMC258946

[B56] Watts E. Zhao Y. Dhara A. Eller B. Patwardhan A. Sinai A. P. (2015). Novel approaches reveal that *Toxoplasma gondii* bradyzoites within tissue cysts are dynamic and replicating entities *in vivo* . mBio 6, e01155–e01115. doi: 10.1128/mBio.01155-15 26350965 PMC4600105

[B57] Weiss L. M. Kim K. (2000). The development and biology of bradyzoites of Toxoplasma gondii. Front. Biosci. 5, D391–D405. doi: 10.2741/weiss 10762601 PMC3109641

[B58] Weitberg A. B. Alper J. C. Diamond I. Fligiel Z. (1979). Acute granulomatous hepatitis in the course of acquired toxoplasmosis. N. Engl. J. Med. 300, 1093–1096. doi: 10.1056/nejm197905103001907 431613

[B59] Zhang Y. W. Halonen S. K. Ma Y. F. Wittner M. Weiss L. M. (2001). Initial characterization of CST1, a Toxoplasma gondii cyst wall glycoprotein. Infect. Immun. 69, 501–507. doi: 10.1128/iai.69.1.501-507.2001 11119543 PMC97909

[B60] Zhu J. Wang Y. Cao Y. Shen J. Yu L. (2021). Diverse roles of tgMIC1/4/6 in the toxoplasma infection. Front. Microbiol. 12. doi: 10.3389/fmicb.2021.666506 PMC8247436 34220751

