# Supplementary figures and images for: Toxoplasma gondii chitinase-like protein TgCLP1 regulates the parasite cyst burden
Source: Front Cell Infect Microbiol. 2024 May 17;14:1359888. doi: 10.3389/fcimb.2024.1359888 (PMC11140023; doi:10.3389/fcimb.2024.1359888)

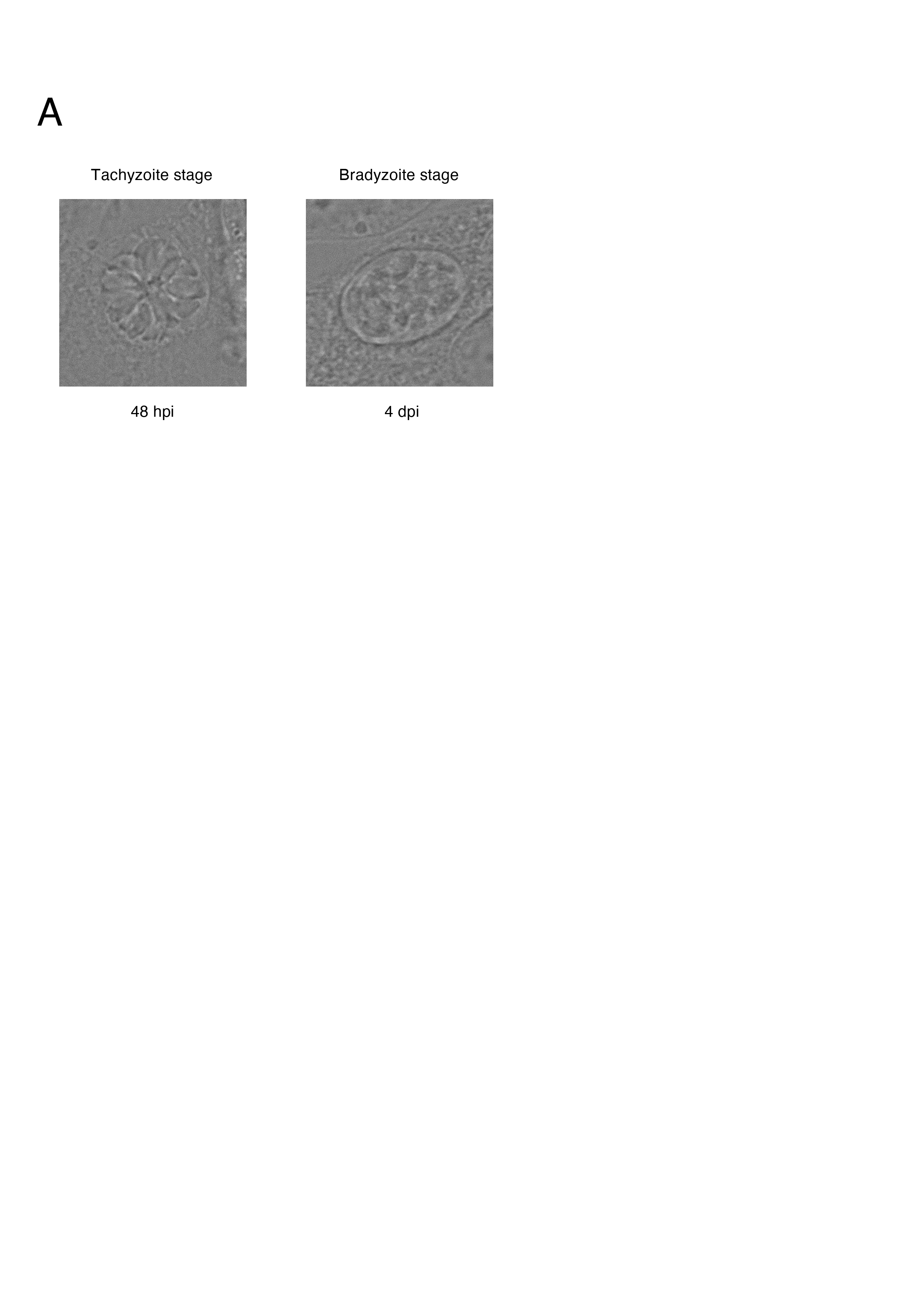

Supplement: Supplementary Figure 1 — Images of parasitophorous vacuole. (A) Representative images of parasitophorous vacuole in tachyzoite or bradyzoite stage. [file Image_1.jpg]

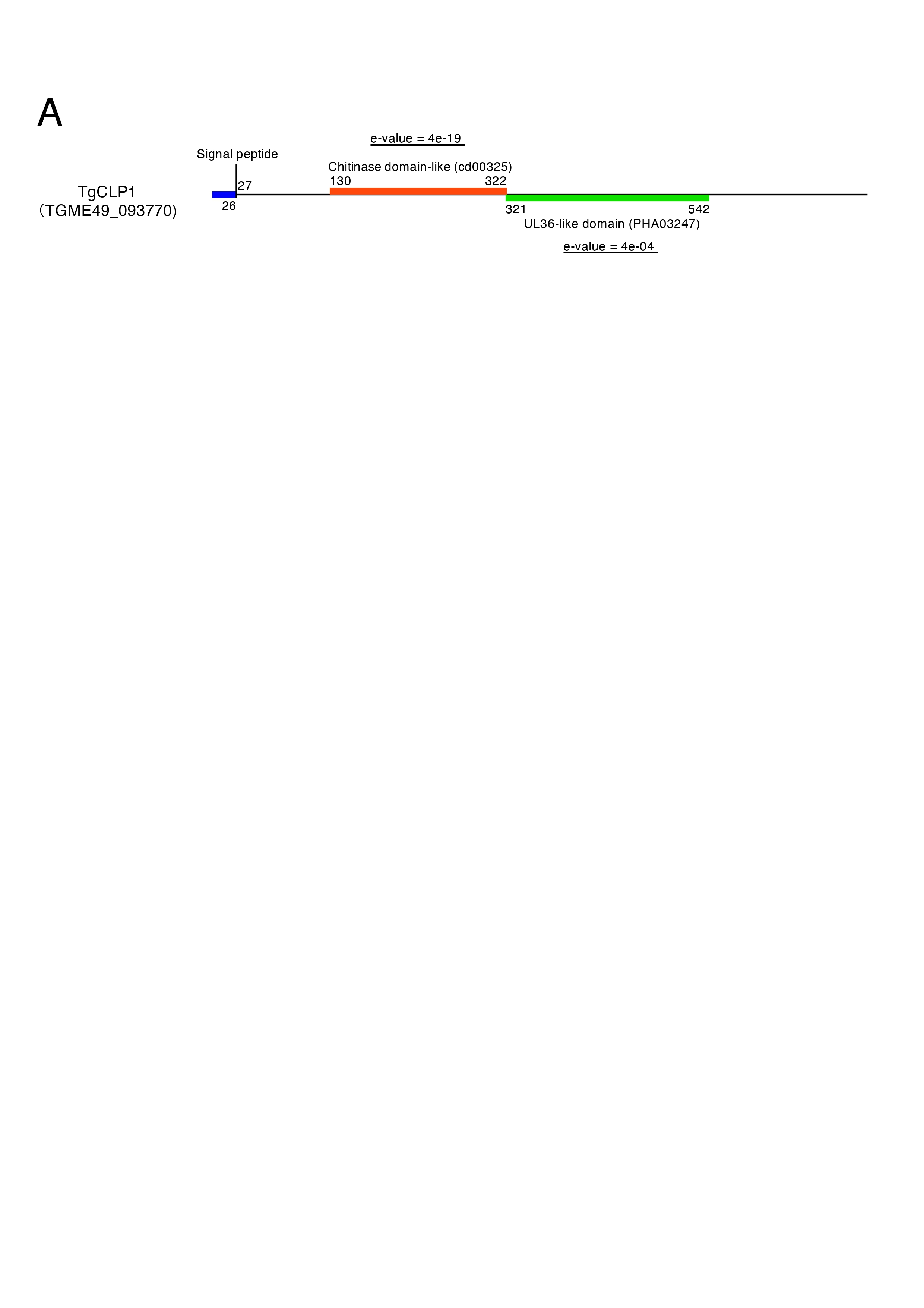

Supplement: Supplementary Figure 2 — Sequence analysis of TgCLP1. (A) Organization of the TgCLP1 sequence. CLP1 domain analyses were performed by ToxoDB, CDD/SPARCLE, and SignalP 5.0. Blue: Signal peptide; residues 1–26. Red: Chitinase domain-like (cd00325); residues 130–322, e-value = 4e-19. Green: large tegument protein UL36-like (PHA03247); residues 321–542, e-value = 4e-04. E-values were calculated by CDD. [file Image_2.jpg]

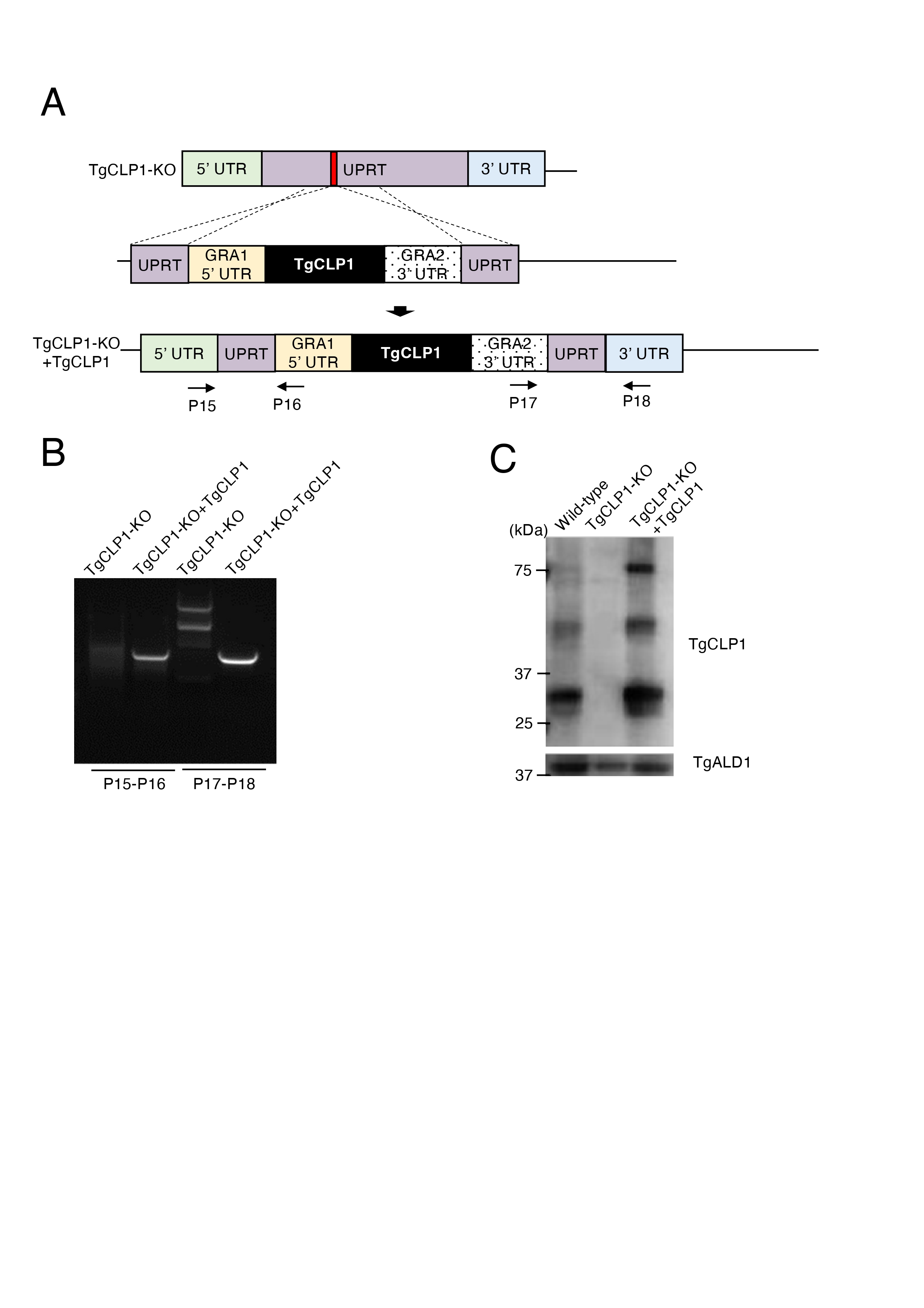

Supplement: Supplementary Figure 3 — Complementation of TgCLP1 knockout parasites. (A) Scheme for generating a TgCLP1 complement in TgCLP1 knockout T. gondii using PruΔku80Δhxgprt LDH2-GFP strain. The construct for inserting the wild-type TgCLP1 gene was placed into the genomic sequence of the UPRT gene through single-homologous recombination. The red bar in UPRT gene represents the region targeted by the sgRNA. (B) The insertion of the wild-type TgCLP1 with the HA tag was verified by PCR with specific primers (P15-P18) of inserting sequence. The complementation was verified by performing a PCR using specific primers (P15-P18) shown in Table S1 and designed for the inserted sequence. (C) Scheme for generating a TgCLP1 knockout T. gondii using PruΔku80Δhxgprt LDH2-GFP strain. The construct for inserting the HXGPRT gene was integrated into the genomic sequence of the TgCLP1 gene through single-homologous recombination. The red bar in TgCLP1 gene represents the region targeted by the sgRNA. The recombination was verified by western blotting. The expression of TgCLP1 was detected in the parasite lysates by western blotting. Parasite-specific ALD1 was used as a loading control. TgCLP1-KO, TgCLP1 knockout T. gondii; TgCLP1-KO+TgCLP1, wild-type TgCLP1 complemented. [file Image_3.jpg]

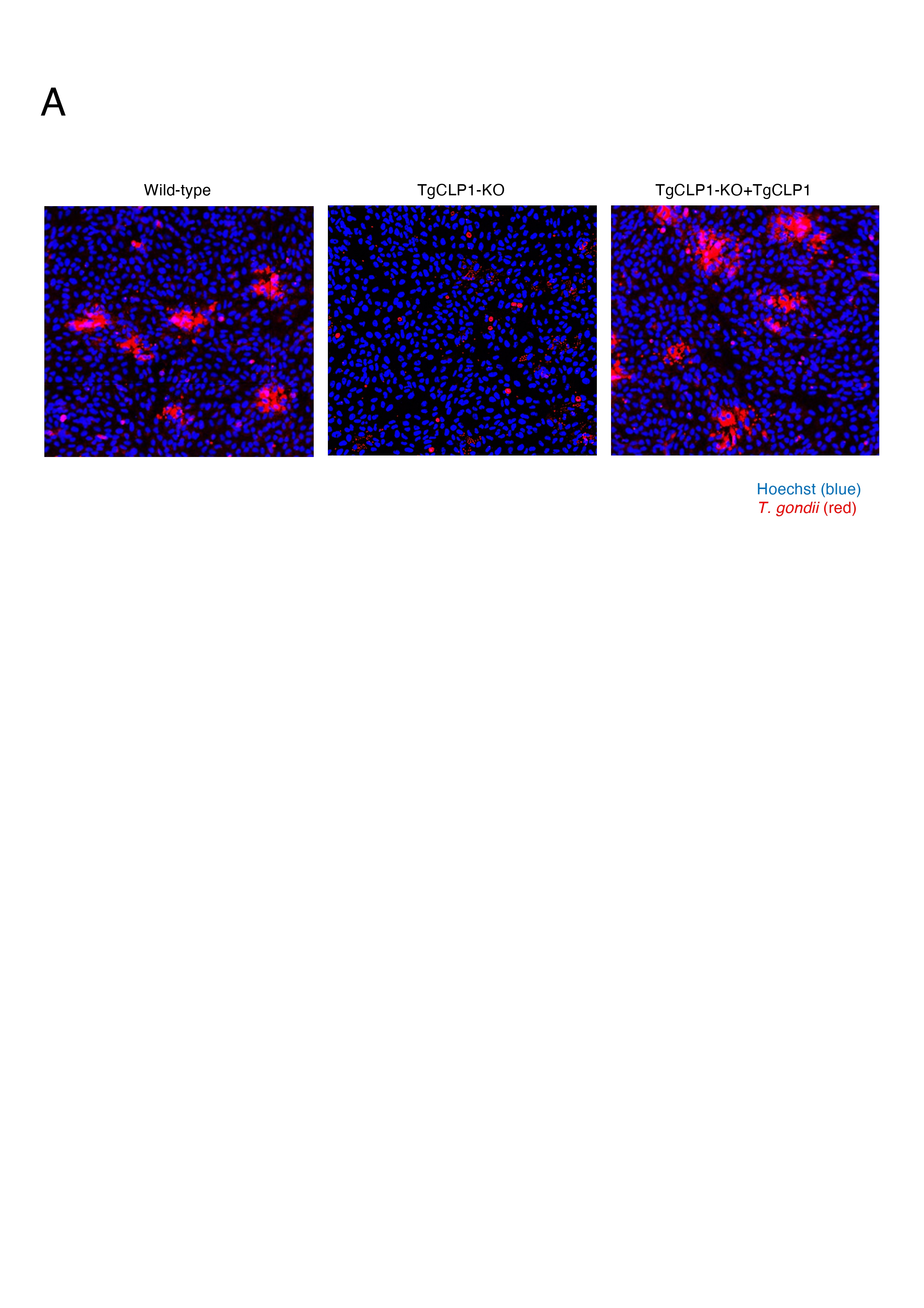

Supplement: Supplementary Figure 4 — Observation of parasite egress. (A) HFF cells were infected with Wild-type, TgCLP1-KO, or TgCLP1-KO+TgCLP1 T. gondii. At 3 days post-infection, parasite expansion was observed by use of fluorescence microscopy. Representative images are provided in the figure. Hoechst, nucleus (blue); RFP, tachyzoite (green). [file Image_4.jpg]

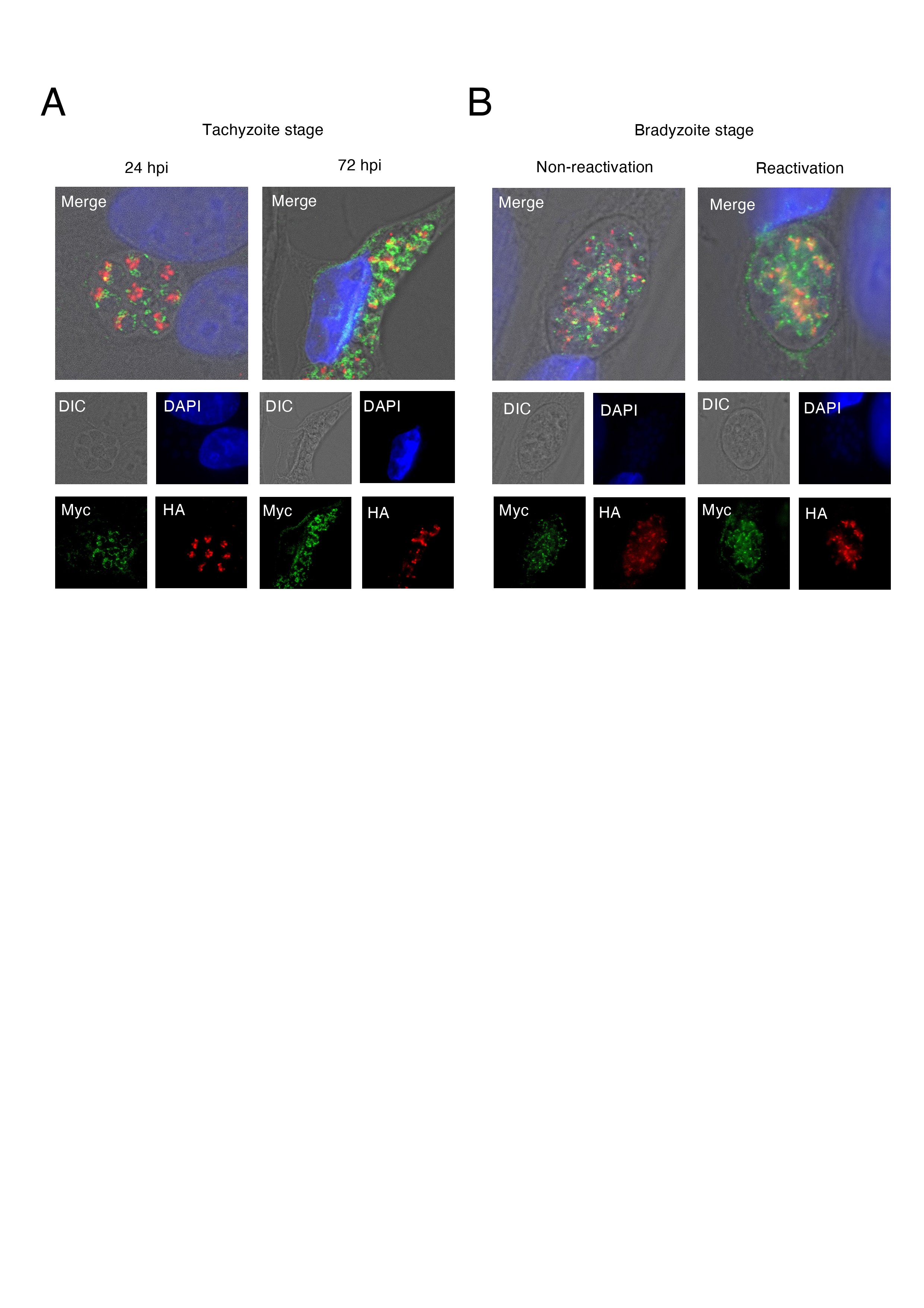

Supplement: Supplementary Figure 5 — Time-resolved localization of TgCLP1. (A, B) T. gondii with N-terminal Myc and C-terminal HA-tagged TgCLP1 was incubated in normal medium (for tachyzoites) or induction medium (for bradyzoites). The expression of Myc-tagged protein or HA-tagged protein was detected by IFA. DAPI, nucleus (blue); Myc, Myc-tagged protein (green); HA, HA-tagged protein (red). (A) At 24 or 72 hours post-infection in the normal medium, tachyzoite stage parasites were observed. (B) At 4 days post-infection in the induction medium, bradyzoite stage non-activated parasites or activated parasites (12 hours reactivation) were observed. [file Image_5.jpg]
